# Factors influencing the career preferences of medical students and interns: a cross-sectional, questionnaire-based survey from India

**DOI:** 10.3352/jeehp.2019.16.12

**Published:** 2019-05-15

**Authors:** Ruban Anand, Prakash Somi Sankaran

**Affiliations:** Department of Biochemistry, Christian Medical College, Vellore, India; Hallym University, Korea

**Keywords:** Career choice, India, Graduate medical education, Medical students

## Abstract

**Purpose:**

The study aimed to identify the motivational factors and demographic variables influencing the career preferences of medical students and interns in India.

**Methods:**

We conducted a questionnaire-based survey at Christian Medical College, Vellore, India. The participants were 368 of the 460 medical students and interns enrolled at the institution from October 2015 to August 2016. We designed the questionnaire to collect demographic data, students’ preferences for career specialties, and the motivational factors influencing them. Then, we analyzed the influence of these factors and demographic variables on career preferences using regression analysis.

**Results:**

Of the 368 respondents, 356 (96.7%) expressed their intention to pursue a residency program (known as postgraduate courses in India) after the Bachelor of Medicine and Bachelor of Surgery (MBBS) program, and about two-thirds indicated their preference to do so in India. The specialties most preferred by students were general surgery, general medicine (internal medicine), and pediatrics, while the least preferred were anatomy, obstetrics and gynecology, and community medicine (also known as social and preventive medicine). Factor analysis yielded three motivational factors, which we named ‘personal growth,’ ‘professional growth,’ and ‘personal satisfaction’ based on the items loaded in each. The motivational factors were predicted by demographic variables (gender, geographical background, current stage in the MBBS program, and the presence of relatives in the health professions). Demographic variables and the motivational factors also had significant influences on career preferences.

**Conclusion:**

This study provides insights into the motivational factors that influence the career preferences of Indian medical students and interns. A robust longitudinal study would be required to study intra-individual variations in preferences and the persistence of choices.

## Introduction

The number of career specialties in medicine has dramatically increased, parallel to the expansion of knowledge in different fields. Given the numerous and diverse options to choose from, the factors that influence the preferences of medical students and interns in India are not well understood. It is known that even at the time of entering a program, medical students have several preconceived notions about the profession and that there is also a general inclination towards certain specialties [1]. Considering that students are taught under a standard curriculum, the factors that steer them to their career choices require analysis. Some theoretical models have also been proposed to explain the career-related decision-making of medical students [2].

The factors involved in career-related decision-making are also context-specific. One of the reasons for this is that models of medical education and selection into specialty programs vary globally. In India, before students opt for a specialty, they are required to complete an undergraduate degree program (Bachelor of Medicine and Bachelor of Surgery, MBBS). The duration of the MBBS program is 4.5 years, with 4 different stages (referred to I to IV MBBS). Upon successful completion of the MBBS program, medical students undergo a compulsory residential rotatory internship (also referred to as internship) in a hospital setting for a year. Subsequently, medical graduates compete for selection into a residency program (known as postgraduate courses in India) through a national-level examination; the mode of selection for admission to the residency programs has also undergone significant changes over the past several years. Finally, graduates are awarded an MS (Master of Surgery; for surgical specialties) or MD (Doctor of Medicine; for non-surgical specialties) degree upon successful completion of a residency program in India.

The perceptions and career preferences of Indian medical students and interns are not well understood. In an earlier study conducted in India, the preferred specialties were ranked by final-year medical students, but motivational factors were not explored [3]. A study conducted at Kasturba Medical College, Manipal showed that students considered personal interest, job opportunities, and income prospects while choosing a specialty; however, the study did not compare differences across students at various stages of the degree program [4]. A study conducted at University College of Medical Sciences, Delhi compared the preferences and motivational factors among students in different years of the degree program; the authors observed that personal interest, interest due to exposure, the perceived reputation of the specialty, lifestyle, income potential, opportunities to settle abroad, and career progression were some of the significant factors considered by students. Most of the factors did not show statistically significant differences between students of different years. However, women were underrepresented in the study (10% of the study participants) [5].

To address the existing knowledge gap, we sought to gain more insight into the factors that influence the career preferences of medical students and interns in India. Therefore, we aimed to study their specialty preferences; furthermore, the motivational factors influencing career preferences were investigated.

## Methods

We conducted this study at Christian Medical College, Vellore, India between October 2015 and August 2016.

### Ethics statement

The Institutional Review Board approved the study protocol (No. 9630 [OBSERVE] dated September 23, 2015). All participants provided written informed consent. This study is a phase (pilot, cross-sectional, and cohort) of an ongoing research project and the results of the cross-sectional phase are presented here. The cohort phase is likely to be completed in 2023.

### Development of the survey instrument

We followed an existing guideline for developing the questionnaire [6]. We synthesized the items in the questionnaire after a review of the literature. The initial version of the questionnaire contained 18 items (Perception of Medical Students on Career Specialty [PMCS] version 1). We invited faculty members (N=10) from the institution to screen the questionnaire for content. We then invited 10 medical students to test the questionnaire for general readability and comprehension.

Furthermore, we also pilot-tested the questionnaire on another cohort of 63 students (Appendix 2; Supplementary Table 1–3). The final version of the questionnaire after pilot-testing was used for the cross-sectional study (PMCS version 4; see Appendix 1). We tested the overall reliability of motivational items in question 9 and the Cronbach α obtained was 0.74, suggestive of acceptable internal consistency. A leave-one-out analysis excluding individual motivational items showed no significant effects on the overall Cronbach α (Appendix 2).

### Description of the survey instrument

We included instructions for the creation of a unique ID for each participant; this also aided in the anonymization of data (additionally, we extracted the information on the age and gender of the participants before subsequent analysis). We designed items 1–4 to collect demographic information (year of joining MBBS, place of childhood upbringing, curriculum of school education, and details of relatives in health professions), while items 5 and 6 inquired about students’ plans for future studies and work. Item 7 inquired about self-reported awareness and certainty regarding the choice(s). Item 8 inquired about specialty preferences; the participants ranked the three most-likely choices in order of their preference and indicated the three least-likely choices without a ranking order. We provided a list of 20 motivational items in item 9 and asked the participants to assign a score of the importance of each item on a 5-point scale (1, not important; 5, very important). In item 10, participants were asked to indicate the time during their degree program when they expected to decide on a career specialty. Items 11 and 12 specifically inquired about participants’ willingness to serve in rural areas.

### Study participants

We surveyed 460 medical students and interns who were enrolled in the MBBS program (I to IV MBBS) at our institution during the study period. All participants had been admitted to the program following a national-level entrance examination followed by interviews at the institution during their entrance year. We organized separate contact sessions for the medical students in a classroom setting; for interns, we administered the survey at their hospital posting sites. Before the survey, we briefed the participants about the purpose of the study and clarified any queries. We also anticipated that a proportion of students might not be of the aware of the available specialties, so we provided a glossary sheet that provided a brief description of each specialty with the questionnaire. Participants were required to complete the paper questionnaires by hand. For participants who missed the contact sessions, we sent 3 reminders (through their respective class representatives). No modes of coercion were used, and the respondents were not pursued further.

### Statistical analysis

All statistical analyses were done using SPSS ver. 16.0 (SPSS Inc., Chicago, IL, USA). We excluded incomplete forms (n=4, 1.1%) from the analysis. The frequency of the distribution of variables between groups was compared using the chi-square test. We performed an initial exploratory factor analysis to reduce the dimensionality, followed by extraction using principal component analysis with varimax rotation and Kaiser normalization. The threshold for factor loading was fixed at 0.45. Factors were selected after examination of the scree plot, with eigenvalues >1. We constructed multivariate regression models to analyze the influence of demographic variables on motivation factors; for these models, female gender, rural background, and not having a family member in the health professions were used as reference categories. All regression models were constructed using the ‘enter’ model.

## Results

**Fig. 1.**
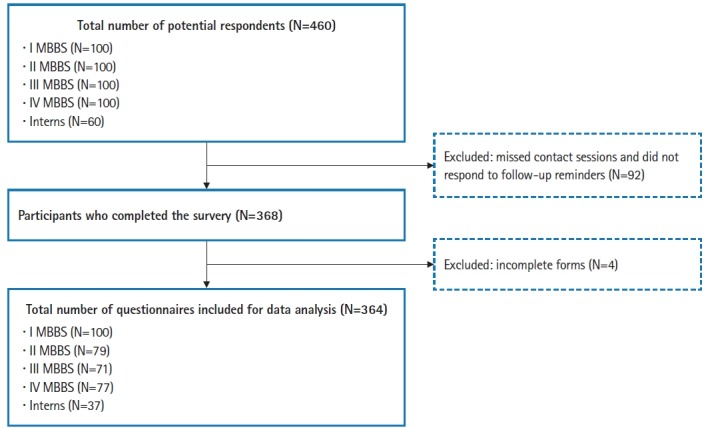
The flow of participants in the study. MBBS, Bachelor of Medicine and Bachelor of Surgery.

Of the 460 participants, 337 medical students and 31 interns completed the survey (Fig. 1). The age of the participants ranged from 17 to 24 years. The distribution of participants based on demographic characteristics (gender, geographic background, and presence of a relative in the health professions) was similar across the different stages of the degree program (Table 1). Overall, most participants (96.7%) expressed their intention to pursue a residency program after receiving their MBBS and about two-thirds intended to do so in India (Table 2). The preferences regarding work settings were not different across the various stages of the degree program. The self-reported awareness and certainty of choices were significantly higher among interns and students in III and IV MBBS (P<0.0001) (Table 2). When explicitly asked, 76.6% of participants indicated their willingness to serve in rural areas. The raw data are available in Supplement 1.

### Motivational factors

Factor analysis loaded the items into 3 factors that explained 50.4% of the total variance. A Kaiser-Mayer-Olkin test statistic of 0.8 was obtained, indicating that the sampling was adequate. We excluded the items (interest in specialty and family members in the same specialty) that did not load onto the 3 factors. We named the extracted factors ‘personal growth,’ ‘professional growth,’ and ‘personal satisfaction’ based on the items loaded in each (Table 3). The factors extracted were found to be reliable (as assessed by Cronbach α values) and valid (as assessed by the factor loadings). We also then analyzed the effect of demographic variables on motivational factors. We found that men rated professional growth significantly higher, while students from an urban background rated personal growth higher. Among participants in the later stages of the degree program, the ratings for personal growth and personal satisfaction were higher, while those for professional growth were lower (Table 4).

### Preferences for specialty

Overall, general surgery, general medicine (also known as internal medicine), and pediatrics were the most preferred specialties (Table 5). The least preferred specialties (Table 6) were anatomy, obstetrics and gynecology, and community medicine (also known as social and preventive medicine). We observed that the preferences became more diverse among participants in the later stages of the degree program. With progression through the program, there was an increased interest in certain specialties (pathology, radiodiagnosis [also known as radiology], forensic medicine, and psychiatry) and for a few others, there was a decline (general surgery, general medicine, pediatrics, pharmacology, physical medicine and rehabilitation [also known as rehabilitation medicine]).

### Influence of variables on preferences for specialty

The specialty preferences showed significant gender bias. Men preferred general surgery and orthopedics, while women preferred pediatrics and obstetrics and gynecology (Table 7). Preferences for pathology and radiodiagnosis increased among participants in the later stages of the degree program, while there was a decrease in preferences for psychiatry and forensic medicine. Participants with a family member in the health professions preferred general medicine and were less likely to prefer forensic medicine. Participants from an urban background were less likely to prefer anesthesia and otorhinolaryngology. We also observed that the ratings of motivational factors had significant influences on specialty preferences (Table 7). Of note, higher ratings for personal growth were associated with a lower preference for general surgery and general medicine. In contrast, higher ratings for professional growth were associated with a greater preference for general medicine and a lower preference for family medicine and community medicine. Ratings for personal satisfaction predicted a greater preference for community medicine, pediatrics, and obstetrics and gynecology.

## Discussion

In this study, we analyzed the preferences of medical students and interns for career specialties and the motivational factors influencing them. Furthermore, we studied differences in preferences among participants at different stages of the degree program. We noted that most students in our study indicated a preference to pursue a residency program in the future, similar to earlier reports from India [3-5]. Additionally, the proportion of students in our study who indicated a preference for pursuing their postgraduate training in India or other countries was similar to those reported in other studies [3,4]. Of note, the preferred specialties in our study (general surgery, general medicine, pediatrics, obstetrics and gynecology, and orthopedics) agree with earlier reports from India and elsewhere [3-5,7]. We also observed that participants in the later stages of the degree program differed in their preferences and motivational factors. Other investigators have also made similar observations [8], suggesting that experiences during the degree program play a significant role in modifying motivational factors.

Although the preferences diversified in the later stages of the degree program, we found that students strongly preferred a select few specialties over others; furthermore, 37.5% of students were unaware of the other options available. The influence of gender on specialty preferences [3-5,9] and motivational factors [10,11] also matched the reports available in the literature. However, there was significant gender bias in the choices. These facts highlight the importance of considering a structured career orientation program in the curriculum. Such a program may increase students’ awareness of the scope and realities, while simultaneously clarifying students’ preferences about various specialties and helping the students to make an informed choice that suits their interests and abilities. Our findings also provide useful information about the specialties that are less preferred by students. Such specialties may need to intensify measures to kindle students’ interest and attract talent. Meanwhile, specialties with strong gender gendered-preferences may need to take measures to allay misconceptions, because doing so will help to ensure gender diversity and inclusivity in the workplace. While the gender-specific preferences in our study agree with the patterns observed elsewhere, our findings are contradictory to a few observations of specialty preferences being similar between men and women [12,13] and findings suggesting that the gender differences may no longer persist after graduation [14,15]. Further studies are required to investigate the persistence of gender-specific preferences beyond graduation in students.

We also noted that 82 students (25.0%) indicated that they would be willing to work in rural areas if given an opportunity (Table 2). However, interest in working in rural areas also showed a declining trend with progression through the course (odds ratio [OR], 0.569; 95% confidence interval [CI], 0.301–1.074). Hence, there is also a need for curricular interventions to sustain students’ interest in working in rural areas. Furthermore, participants motivated by personal satisfaction were more likely to prefer working in rural areas (OR, 18.01; 95% CI, 4.5–72.01; P<0.001). Hence, identifying these individuals during the admission process or the degree program may help to increase the number of doctors entering service in rural areas.

Our findings also show that 233 respondents (63.5%) had a relative in the health professions. Interestingly, the motivational patterns and preferences of those students were different from those of students who did not have a relative in the health professions, and they were also less likely to prefer rural service. The influence of the presence of a family member in the health professions on motivational factors needs to be studied further. In addition, the finding that most of our study participants were from an urban background is another statistic that needs to be considered seriously from a policy perspective. More measures need to be implemented to ensure increased representation of students from a rural background.

### Strengths and limitations

A strength of our study is the inclusion of interns and students at each stage of the medical degree program. We also used a questionnaire designed and standardized to the local setting, and reliability testing showed high internal consistency. Limitations of the study include its cross-sectional design and the fact that it only analyzed students’ preferences during the undergraduate medical program, but not their actual choices. Whether the participants would persist with their indicated choice and commit to it in the future was not studied. Furthermore, 20% of potential respondents (including 38.3% of the interns) did not participate in the survey. These limitations need to be borne in mind while interpreting the findings of our study.

### Future directions

A robust longitudinal study is required to study intra-individual variations in preferences and the persistence of choices among students. Following up the students until they make a final choice may also help to answer the question of whether the factors analyzed in this study can predict the actual choice of specialty. We are currently following up a student cohort (MBBS entering class of 2016) to study these aspects.

This study provides insights into the career preferences of Indian medical students and interns and the underlying motivational factors. The findings from this study suggest the need for a structured career-orientation program at the institutional level that would guide the students in making their career choices. We also found differences in these variables due to participants’ characteristics. The findings of our study also suggest a topic for debate: should admission to medical schools be purely meritocratic (as is the current practice) or are other background variables (such as gender, geographical background, and whether a prospective student has a relative in the health professions) essential too?

## Figures and Tables

**Table 1.** Demographic characteristics of the study participants

Characteristic	Total	I MBBS	II MBBS	III MBBS	IV MBBS	Interns	χ^2^	df	P-value
Gender							2.211	4	0.697
Men	138 (37.9)	42 (42.0)	29 (36.7)	25 (35.2)	31 (40.3)	11 (29.7)			
Women	226 (62.1)	58 (58.0)	50 (63.3)	46 (64.8)	46 (59.7)	26 (70.3)			
Geographical background							4.366	4	0.359
Rural	50 (13.7)	18 (18.2)	7 (9.0)	12 (17.0)	8 (9.9)	4 (11.1)			
Urban	316 (86.3)	81 (81.8)	71 (91.0)	59 (83.0)	73 (90.1)	32 (88.9)			
Relatives in the health professions							6.153	4	0.188
No	134 (36.5)	32 (32.0)	37 (46.8)	30 (38.0)	25 (30.9)	13 (35.1)			
Yes	233 (63.5)	68 (68.0)	42 (53.2)	49 (62.0)	56 (69.1)	24 (64.9)			

Values are presented as number (%). MBBS, Bachelor of Medicine and Bachelor of Surgery; df, degrees of freedom.

**Table 2.** Career intentions and workplace preferences of the study participants

Variable	Total	I MBBS	II MBBS	III MBBS	IV MBBS	Interns	χ^2^	df	P-value
Item 5-(1)							13.4	8	0.099
Yes	356 (96.7)	95 (95.0)	76 (96.2)	68 (95.8)	81 (100.0)	36 (97.3)			
No	2 (0.5)	-	-	2 (2.8)	-	-			
Not sure	10 (2.7)	5 (5.0)	3 (3.8)	1 (1.4)	-	1 (2.7)			
Item 5-(2)							17.3	8	0.027
India	213 (60.2)	41 (43.2)	49 (65.3)	45 (66.2)	53 (66.2)	25 (69.4)			
Abroad	57 (16.1)	24 (25.3)	10 (13.3)	7 (10.3)	11 (13.8)	5 (13.9)			
Not sure	84 (23.7)	30 (31.5)	16 (21.3)	16 (23.5)	16 (20.0)	6 (16.7)			
Item 6-(1)							14.3	8	0.075
Urban areas	118 (36.0)	25 (28.7)	24 (33.8)	24 (37.5)	34 (45.3)	11 (35.5)			
Rural areas	82 (25.0)	19 (21.8)	16 (22.5)	22 (34.4)	14 (18.7)	11 (35.5)			
Not sure	128 (39.0)	43 (49.4)	31 (43.7)	18 (28.1)	27 (36.0)	9 (29.0)			
Item 6-(2)							9.8	12	0.639
Mission hospital	115 (40.1)	25 (34.2)	24 (36.4)	26 (43.3)	25 (39.1)	15 (62.5)			
Tertiary hospital	126 (43.9)	34 (46.6)	32 (48.5)	25 (41.7)	29 (45.3)	6 (25.0)			
Own or private hospital	33 (11.5)	9 (12.3)	6 (9.1)	7 (11.7)	8 (12.5)	3 (12.5)			
Others	13 (4.5)	5 (6.9)	4 (6.0)	3 (3.3)	2 (3.1)	-			
Item 7							52.3	12	<0.0001
Not aware, not certain	138 (37.7)	51 (51.5)	40 (51.3)	15 (21.1)	25 (30.9)	7 (18.9)			
Not aware, certain	82 (22.4)	30 (30.3)	16 (20.5)	12 (16.9)	16 (19.8)	8 (21.6)			
Aware, not certain	115 (31.4)	16 (16.2)	18 (23.1)	33 (46.5)	32 (39.5)	16 (43.2)			
Aware, certain	31 (8.5)	2 (2.0)	4 (5.1)	11 (15.5)	8 (9.8)	6 (16.2)			

Values are presented as number (%). Item 5-(1): Are you planning to do a postgraduate degree after MBBS? Item 5-(2): If your response was “yes,” where would you like to do your postgraduate degree after MBBS? Item 6-(1): Where would you prefer to work after completion of your postgraduate degree? Item 6-(2): Specific work setting. Item 7: Which of the following statements best describe your current state of mind, regarding (1) the awareness on the various career specialties available? and (2) the certainty of the course you want to pursue? MBBS, Bachelor of Medicine and Bachelor of Surgery; df, degrees of freedom.

**Table 3.** Factors influencing the career preferences of study participants

Factors: items	Factor loadings			Eigenvalue	% Variance explained	Cronbach α
	1	2	3			
Personal growth				3.97	22.08	0.840
Flexible working hours	0.839					
Less stressful working conditions	0.838					
Less duration of work hours	0.767					
Comfortable lifestyle	0.739					
Sufficient time for hobbies and personal interests	0.720					
Family responsibilities	0.519					
Professional growth				2.62	14.6	0.694
Opportunities for higher studies or further specialization		0.805				
Opportunity to do research		0.645				
Perceived status of the field		0.584				
Opportunity to settle down in urban areas		0.547				
Professionally challenging		0.528				
Financial prospects		0.487				
Personal satisfaction				2.47	13.7	0.668
Opportunity to be involved in patient care			0.675			
Preference to work in rural areas			0.649			
Opportunity to teach			0.600			
Influences from past experiences			0.579			
Influenced by role models			0.569			
Sense of calling			0.554			
Items excluded from factor analysis						
Interest in a specialty						
Family member in the same specialty						

We analyzed the motivational items through exploratory factor analysis, followed by extraction by principal component analysis with varimax rotation and Kaiser normalization. We fixed the threshold for factor loading at 0.45. After examination of the scree plot, we selected the factors with eigenvalues >1. A Kaiser-Mayer-Olkin test statistic of 0.8 was obtained, indicating that sampling was adequate.

**Table 4.** Influence of demographic variables on the factors affecting the career preferences of study participants

Dependent variable	Gender (ref: female)	Geographical background (ref: rural)	Relative in the health professions (ref: no relative)	Current stage in MBBS (ref: I MBBS)	Regression model parameters		
					R^2^	F statistic	P-value
Personal growth	0.06 (-0.15 to 0.28)	0.33 (0.02 to 0.64)^*^	0.21 (-0.003 to 0.04)^****^	0.15 (0.07 to 0.23)^***^	0.067	6.12	<0.0001
Professional growth	0.33 (0.11 to 0.55)^*^	0.18 (-0.13 to 0.49)	-0.25 (-0.47 to -0.03)^*^	-0.08 (-0.16 to -0.002)^*^	0.052	4.67	0.001
Personal satisfaction	-0.2 (-0.42 to 0.02)	-0.19 (-0.5 to 0.12)	-0.13 (-0.35 to 0.09)	0.11 (0.03 to 0.19)^**^	0.038	3.340	0.011

Values are presented as regression coefficient (95% confidence interval). We constructed multivariate linear regression models to study the predictors for motivational factors using standardized z-scores calculated for each factor. For all regression models, female gender, rural background, and not having a relative in the health professions were the reference categories, and the ‘enter’ model was used. Unstandardized regression coefficients are shown. Ref, reference; MBBS, Bachelor of Medicine and Bachelor of Surgery.

* P<0.05,

** P<0.01,

*** P<0.001,

**** P<0.06.

**Table 5.** Frequencies of ‘most likely’ choices as indicated by the participants

Specialty	Total	I MBBS	II MBBS	III MBBS	IV MBBS	Interns
Anatomy	9 (0.8)	2 (0.7)	1 (0.4)	4 (1.9)	1 (0.4)	1 (0.9)
Anesthesia	26 (2.4)	9 (3.2)	4 (1.7)	1 (0.5)	12 (5.0)	-
Biochemistry	4 (0.4)	3 (1.1)	1 (0.4)	-	-	-
Community medicine	28 (2.6)	7 (2.5)	8 (3.4)	4 (1.9)	4 (1.7)	5 (4.5)
Dermatology	20 (1.9)	5 (1.8)	3 (1.3)	4 (1.9)	7 (2.9)	1 (0.9)
Emergency medicine	49 (4.5)	11 (3.9)	12 (5.1)	8 (3.9)	14 (5.8)	4 (3.6)
Family medicine	26 (2.4)	2 (0.7)	3 (1.3)	7 (3.4)	9 (3.8)	5 (4.5)
Forensic medicine	18 (1.7)	13 (4.6)	3 (1.3)	1 (0.5)	-	1 (0.9)
General medicine	200 (18.5)	33 (11.6)	64 (27.1)	31 (15.0)	50 (20.8)	22 (19.8)
General surgery	203 (18.8)	58 (20.4)	48 (20.3)	43 (20.8)	39 (16.2)	15 (13.5)
Geriatric medicine	8 (0.7)	-	3 (1.3)	1 (0.5)	2 (0.8)	2 (1.8)
Microbiology	4 (0.4)	2 (0.7)	-	1 (0.5)	-	1 (0.9)
Nuclear medicine	12 (1.1)	7 (2.5)	3 (1.3)	1 (0.5)	-	1 (0.9)
Obstetrics and gynecology	61 (5.7)	20 (7.0)	11 (4.7)	10 (4.8)	14 (5.8)	6 (5.4)
Ophthalmology	2.4 (26)	9 (3.2)	6 (2.5)	5 (2.4)	5 (2.1)	3 (2.7)
Orthopedics	52 (4.8)	13 (4.6)	24 (10.2)	14 (6.8)	13 (5.4)	6 (5.4)
Otorhinolaryngology	27 (2.5)	10 (3.5)	2 (0.8)	2 (1.0)	10 (4.2)	3 (2.7)
Pathology	12 (1.1)	2 (0.7)	-	4 (1.9)	1 (0.4)	5 (4.5)
Pediatrics	129 (12.0)	38 (13.3)	24 (10.2)	39 (18.8)	15 (6.2)	13 (11.7)
Pharmacology	6 (0.6)	3 (1.1)	1 (0.4)	-	2 (0.8)	-
Physical medicine and rehabilitation	13 (1.2)	5 (1.8)	4 (1.7)	2 (1.0)	1 (0.4)	1 (0.9)
Physiology	9 (0.8)	2 (0.7)	2 (0.8)	2 (1.0)	2 (0.8)	2 (1.8)
Psychiatry	42 (3.9)	16 (5.6)	9 (3.8)	9 (4.3)	7 (2.9)	1 (0.9)
Radiodiagnosis	40 (3.7)	1 (0.4)	4 (1.7)	9 (4.3)	17 (7.1)	9 (8.1)
Radiotherapy	19 (1.8)	9 (3.2)	3 (1.3)	2 (1.0)	5 (2.1)	-
Respiratory medicine	10 (0.9)	1 (0.4)	4 (1.7)	1 (0.5)	4 (1.7)	-
Transfusion medicine	3 (0.3)	-	-	2 (1.0)	1 (0.4)	-
Not sure	7 (0.6)	1 (0.4)	1 (0.4)	1 (0.5)	2 (0.8)	2 (1.8)
Total	1063	285	236	207	240	109

Values are presented as number (%). Each participant indicated 3 choices as most likely. The cumulative frequencies are shown in the table. Question: item 8-(1): The broad medical specialty degrees (MD, MS, DNB) that a doctor can choose after MBBS are listed. Indicate 3 specialties you would most likely choose. MBBS, Bachelor of Medicine and Bachelor of Surgery; MD, Doctor of Medicine; MS, Master of Surgery; DNB, Diplomate in National Board.

**Table 6.** Frequencies of ‘least likely’ choices as indicated by the participants

Specialty	Total	I MBBS	II MBBS	III MBBS	IV MBBS	Interns
Anatomy	96 (8.9)	29 (10.2)	21 (8.9)	18 (8.7)	16 (6.7)	12 (10.8)
Anesthesia	28 (2.6)	13 (4.6)	3 (1.3)	6 (2.9)	2 (0.8)	4 (3.6)
Biochemistry	65 (6.0)	24 (8.4)	10 (4.2)	15 (7.2)	9 (3.8)	7 (6.3)
Community medicine	81 (7.5)	5 (1.9)	25 (10.6)	15 (11.1)	22 (9.2)	6 (5.4)
Dermatology	48 (4.4)	15 (5.3)	4 (1.7)	17 (8.2)	8 (3.3)	4 (3.6)
Emergency medicine	8 (0.7)	2 (0.7)	2 (0.8)	-	2 (0.8)	2 (1.8)
Family medicine	19 (1.8)	4 (1.4)	7 (3.0)	3 (1.4)	3 (1.2)	2 (1.8)
Forensic medicine	55 (5.1)	4 (1.4)	11 (4.7)	16 (7.7)	15 (6.2)	9 (8.1)
General medicine	16 (1.5)	1 (0.4)	2 (0.8)	3 (1.4)	5 (2.1)	5 (4.5)
General surgery	24 (2.2)	3 (1.1)	14 (1.7)	3 (1.4)	8 (3.3)	6 (5.4)
Geriatric medicine	17 (1.6)	3 (1.1)	5 (2.1)	4 (1.9)	3 (1.2)	2 (1.8)
Microbiology	53 (4.9)	9 (3.2)	21 (8.9)	4 (1.9)	15 (6.2)	4 (3.6)
Nuclear medicine	24 (2.2)	11 (3.9)	3 (1.3)	3 (1.4)	5 (2.1)	2 (1.8)
Obstetrics and gynecology	85 (7.9)	20 (7.0)	15 (6.4)	14 (6.8)	24 (10.0)	12 (10.8)
Ophthalmology	24 (2.2)	6 (2.1)	7 (3.0)	4 (1.9)	4 (1.7)	3 (2.7)
Orthopedics	37 (3.4)	7 (2.5)	3 (1.3)	9 (4.3)	13 (5.4)	5 (4.5)
Otorhinolaryngology	18 (1.7)	2 (2.8)	1 (0.4)	2 (1.0)	5 (2.1)	2 (1.8)
Pathology	44 (4.1)	12 (4.2)	17 (7.2)	6 (2.9)	9 (3.8)	-
Pediatrics	21 (1.9)	5 (1.8)	2 (0.8)	4 (1.9)	8 (3.3)	2 (1.8)
Pharmacology	70 (6.5)	15 (5.3)	18 (7.6)	15 (7.2)	20 (8.3)	2 (1.8)
Physical medicine and rehabilitation	29 (2.7)	10 (3.5)	7 (3.0)	6 (2.9)	4 (1.7)	2 (1.8)
Physiology	23 (2.1)	9 (3.2)	6 (2.6)	7 (3.4)	-	1 (0.9)
Psychiatry	46 (4.3)	11 (3.9)	8 (3.4)	12 (5.8)	11 (4.6)	4 (3.6)
Radiodiagnosis	15 (1.4)	3 (1.1)	7 (3.0)	3 (1.4)	2 (0.8)	-
Radiotherapy	21 (1.9)	9 (3.2)	5 (2.1)	-	-	7 (6.3)
Respiratory medicine	5 (0.5)	4 (1.4)	-	1 (0.5)	-	-
Transfusion medicine	15 (1.4)	8 (2.8)	4 (1.7)	3 (1.4)	-	-
Not sure	-	-	-	-	-	-
Total	987	250	218	201	213	105

Values are presented as number (%). Each participant indicated three choices as least likely. The cumulative frequencies are shown in the table. Question: item 8-(2): The broad medical specialty degrees (MD, MS, DNB) that a doctor can choose after MBBS are listed below. Indicate three specialties you would least likely choose MBBS, Bachelor of Medicine and Bachelor of Surgery; MD, Doctor of Medicine; MS, Master of Surgery; DNB, Diplomate in National Board.

**Table 7.** Influence of demographic variables and motivational factors on the specialty preferences of the participants

Specialty	Logistic regression models for most likely choice (vs. least likely choice)						
	Gender	Geographical background	Relative in the health professions	Current stage in MBBS	Personal growth	Professional growth	Personal satisfaction
Anatomy	0.63 (0.11–3.53)	0.68 (0.07–6.97)	0.713 (0.15–3.37)	0.86 (0.47–1.6)	1.27 (0.56–2.85)	0.74 (0.33–1.66)	0.57 (0.57–2.73)
Anesthesia	1.72 (0.43–6.92)	0.19^*^ (0.05–0.8)	1.35 (0.25–7.27)	1.38 (0.84–2.28)	1.65 (0.84–3.23)	0.5 (0.22–1.14)	0.38^*^ (0.17–0.88)
Biochemistry	12.34 (0.6–275.5)	NE	0.826 (0.05–14.14)	0.206 (0.03–1.36)	1.04 (0.36–3.02)	0.93 (0.13–6.69)	2.95 (0.59–14.54)
Community medicine	1.99 (0.61–6.5)	2.37 (0.37–15.34)	2.52 (0.75–8.42)	0.62 (0.34–1.11)	0.62 (0.33–1.19)	0.39^**^ (0.19–0.69)	2.66^*^ (1.26–5.65)
Dermatology	0.17^*^ (0.03–0.92)	0.20 (0.02–2.07)	1.06 (0.25–4.56)	1.06 (0.6–1.87)	2.23^*^ (1.15–4.59)	0.79 (0.4–1.54)	0.56 (0.3–1.07)
Emergency medicine	0.38 (0.5–2.65)	NE	1.3 (0.18–9.57)	1.15 (0.62–2.1)	0.54 (0.22–1.3)	4.54^*^ (1.13–18.32)	0.65 (0.23–1.84)
Family medicine	0.09 (0.08–1.24)	0.27 (0.14–5.3)	1.7 (0.12–26.41)	1.37 (0.49–3.79)	0.42 (0.12–1.45)	0.24^*^ (0.06–0.87)	1.88 (0.57–6.19)
Forensic medicine	1.59 (0.3–8.5)	2.11 (0.09–45.8)	0.142^*^ (0.02–0.9)	0.24^**^ (0.10–0.56)	0.89 (0.36–2.2)	0.8 (0.34–1.88)	1.9 (0.64–5.62)
General medicine	2.5 (0.6–10.49)	1.67 (0.27–10.5)	3.68^*^ (1.12–12.03)	0.79 (0.46–1.33)	0.54^****^ (0.29–1.01)	2.01^*^ (1.11–3.63)	0.98 (0.54–1.77)
General surgery	5.08^*^ (1.3–19.77)	1.69 (0.42–6.81)	1.48 (0.53–4.14)	0.69 (0.46–1.03)	0.33^***^ (0.18–0.62)	1.54 (0.92–2.56)	1.24 (0.75–2.05)
Geriatric medicine	NE	NE	NE	NE	NE	NE	NE
Microbiology	NE	NE	NE	0.85 (0.26–2.84)	2.86 (0.5–16.27)	1.51 (0.16–14.67)	0.48 (0.13–1.86)
Nuclear medicine	0.97 (0.10–9.4)	1.38 (0.15–12.88)	1.56 (0.22–10.87)	0.91 (0.33–2.54)	1.09 (0.35–3.45)	7.48^*^ (1.08–51.41)	0.41 (0.12–1.35)
Obstetrics and gynecology	0.12^***^ (0.04–0.36)	0.49 (0.14–1.64)	0.78 (0.36–1.72)	0.75 (0.55–1.02)	0.87 (0.59–1.26)	0.79 (0.53–1.17)	1.8^**^ (1.17–2.79)
Ophthalmology	0.68 (0.14–3.3)	1.99 (0.27–14.45)	2.36 (0.62–8.97)	1.03 (0.68–1.66)	1.44 (0.71–2.9)	0.99 (0.48–2.03)	0.7 (0.29–1.69)
Orthopedics	21.09^***^ (5.38–82.72)	0.992 (0.19–4.97)	0.54 (0.17–1.72)	0.86 (0.56–1.33)	0.67 (0.36–1.25)	1.46 (0.81–2.65)	1.21 (0.69–2.12)
Otorhinolaryngology	2.16 (0.34–13.77)	0.042^*^ (0.002–0.71)	2.39 (0.41–13.81)	1.4 (0.73–2.68)	2.94^*^ (1.05–8.22)	1.02 (0.47–2.18)	0.74 (0.32–1.69)
Pathology	2.78 (0.36–21.23)	1.94 (0.15–24.71)	0.09 (0.007–1.18)	3.14^*^ (1.23–8.01)	4.01^*^ (1.23–13.08)	0.55 (0.16–1.99)	0.16^*^ (0.03–0.82)
Pediatrics	0.24^**^ (0.08–0.68)	0.71 (0.23–2.22)	0.79 (0.17–3.77)	0.69 (0.45–1.05)	1.43 (0.79–2.59)	0.85 (0.51–1.42)	2.1^*^ (1.18–3.74)
Pharmacology	3.94 (0.32–48.9)	NE	0.18 (0.009–3.72)	0.35^****^ (0.12–1.01)	6.46^*^ (1.38–38.21)	0.63 (0.15–2.66)	1.0 (0.24–4.29)
Physical medicine and rehabilitation	0.04 (0–2.86)	0.21 (0.004–10.57)	4.54 (0.41–50.9)	0.56 (0.19–1.69)	1.74 (0.44–6.84)	0.21^*^ (0.06–0.79)	6.22 (0.86–45.01)
Physiology	13.11 (0.7–258.2)	NE	0.81 (0.04–16.92)	2.87 (0.94–8.82)	0.86 (0.2–3.66)	0.45 (0.12–1.65)	0.27 (0.07–1.14)
Psychiatry	0.77 (0.27–2.16)	2.66 (0.57–12.36)	0.61 (0.19–1.9)	0.65^*^ (0.43–0.98)	1.21 (0.72–2.06)	0.68 (0.41–1.13)	0.45^**^ (0.26–0.78)
Radiodiagnosis	1.32 (0.14–12.1)	3.05 (0.14–65.94)	2.37 (0.29–19.6)	4.89^**^ (1.6–15.34)	2.38 (0.82–6.95)	5.3^*^ (1.39–20.23)	0.69 (0.17–2.72)
Radiotherapy	0.31 (0.06–1.68)	1.37 (0.21–9.19)	2.99 (0.52–17.27)	0.84 (0.49–1.44)	0.96 (0.37–2.48)	1.92 (0.75–4.94)	0.8 (0.41–1.59)
Respiratory medicine	NE	NE	NE	NE	NE	NE	NE
Transfusion medicine	NE	NE	NE	NE	NE	NE	NE

Values are presented as odds ratio (95% confidence interval). We constructed multivariate logistic regression models to study the predictors of specialty, with preference as a dichotomous outcome (participants who indicated the specialty in their most likely choices vs. participants who indicated the specialty in the least likely choices). For all regression models, female gender, rural background, and not having a relative in the health professions were the reference categories, and the ‘enter’ model was used. For each motivational factor, standardized z-scores were calculated and used in the regression analysis. MBBS, Bachelor of Medicine and Bachelor of Surgery; NE, not estimated.

* P<0.05,

** P<0.01,

*** P<0.001,

**** P<0.06.

## Supplementary material

Supplement 1. Data files are available from https://doi.org/10.7910/DVN/5DN39N

Supplement 2. Audio recording of the abstract.

## Appendix 2 Supplementary tables 1-4.

**Supplementary Table 1.** Demographic characteristics and career intentions of the participants of the pilot study

Parameters of interest	Category	No. (%)
Gender	Men	22 (34.9)
	Women	41 (65.1)
Geographical background	Urban	48 (76.2)
	Rural	11 (17.5)
Relatives in the health professions	Yes	41 (65.1)
	No	22 (34.9)
Item 5: Are you planning to do a postgraduate degree after MBBS?	Yes	60 (95.2)
	No	3 (4.8)
Item 5-(1): If your response was “yes,” where would you like to do your postgraduate degree after MBBS?	India	45 (75.0)
	Abroad­­­	5 (8.3)
	Not sure	10 (16.7)
Item 7: Which of the following statements best describe your current state of mind, regarding (1) the awareness on the various career specialties available? and (2) the certainty of the course you want to pursue?	Not aware, not certain	24 (38.1)
	Not aware, certain	10 (15.9)
	Aware, not certain	25 (39.7)
	Aware, certain	4 (6.3)

MBBS, Bachelor of Medicine and Bachelor of Surgery.

**Supplementary Table 2.** Frequencies of the ‘most likely’ and ‘least likely’ specialty choices as indicated by the participants of the pilot study

Specialty	Item 8: The broad medical specialty degrees (MD, MS, DNB) that a doctor can choose after MBBS are listed below.	
	Item 8-(1): Indicate three specialties you would most likely choose	Item 8-(2): Indicate three specialties you would least likely choose
Anatomy	1 (0.5)	10 (5.5)
Anesthesia	1 (0.5)	6 (3.3)
Biochemistry	0	9 (5.0)
Community medicine	3 (1.6)	18 (9.9)
Dermatology	8 (4.3)	13 (7.2)
Emergency medicine	9 (4.8)	4 (2.2)
ENT (ear, nose, or throat)	2 (1.1)	2 (1.1)
Family medicine	5 (2.7)	4 (2.2)
Forensic medicine	2 (1.1)	10 (5.5)
General medicine	30 (16.1)	3 (1.7)
General surgery	33 (17.7)	6 (3.3)
Geriatric medicine	3 (1.6)	3 (1.7)
Microbiology	1 (0.5)	11 (6.1)
Nuclear medicine	1 (0.5)	5 (2.8)
Obstetrics & gynecology	15 (8.1)	8 (4.4)
Ophthalmology	4 (2.2)	1 (0.6)
Orthopedics	14 (7.5)	5 (2.8)
Pathology	2 (1.1)	5 (2.8)
Pediatrics	37 (19.9)	3 (1.7)
Pharmacology	0	21 (11.6)
Physical medicine and rehabilitation	1 (0.5)	4 (2.2)
Physiology	1 (0.5)	4 (2.2)
Psychiatry	7 (3.8)	5 (2.8)
Radiodiagnosis	2 (1.1)	4 (2.2)
Radiotherapy	1 (0.5)	7 (3.9)
Respiratory medicine	2 (1.1)	1 (0.6)
Transfusion medicine	0	7 (3.9)
Not sure	1 (0.5)	2 (1.1)
Total	186	181

Values are presented as number (%). MD, Doctor of Medicine; MS, Master of Surgery; DNB, Diplomate in National Board; MBBS, Bachelor of Medicine and Bachelor of Surgery.

**Supplementary Table 3.** Factors influencing the career preferences of the participants of the pilot study (N=63)

Item 9.	Mean rating±standard deviation	P-values of the comparisons		
		Gender (women vs. men)	Geographical background (rural vs. urban)	Relatives in the health professions (yes vs. no)
Financial prospects	3.03±0.93	0.027^a)^	0.389	0.341
Perceived status of the field in society	3.22±1.17	0.457	1.000	0.677
Opportunities for higher studies or further specialization	3.78±1.13	0.083	0.388	0.851
Professionally challenging field	3.84±1.09	0.085	0.312	0.439
Less stressful working conditions	3.05±1.21	0.503	0.561	0.066
Flexible working hours	3.21±1.17	0.749	0.718	0.035^a)^
Opportunity to settle down in urban areas	2.71±1.13	0.025^a)^	0.301	0.789
Influence from past experiences	3.78±0.99	0.127	0.782	0.897
Opportunity to do research	2.38±1.32	0.079	0.149	0.415
Interest in the specialty	4.78±0.58	0.650	0.744	0.820
Family responsibilities	3.75±1.12	0.491	0.597	0.581
Preference to work in rural areas	3.48±1.15	0.617	0.444	0.394
Opportunity to be involved in patient care	4.41±0.79	0.615	0.903	0.103
Opportunity to teach	3.24±1.20	0.147	0.514	0.503
Less duration of work hours	2.71±1.01	0.821	0.422	0.008^a)^
Influence by role models	3.76±1.17	0.097	0.700	0.615
Sufficient time for hobbies and personal interests	3.30±1.21	0.853	0.536	0.100
Family members in the same specialty	1.63±0.89	0.780	0.393	0.036^a)^
Comfortable lifestyle	2.89±1.03	0.742	0.236	0.005^a)^
Sense of calling	4.11±0.92	0.509	0.003^a)^	0.866

P-values obtained using the Mann-Whitney U-test. Significant P-values are shown in bold. Question: item 9: On a scale of 1 to 5 (where, 1=not important and 5=very important), how would you rate the importance to each of the following factors for choosing a specialty?

a) Significant P-value.

**Supplementary Table 4.** Cronbach α values after the exclusion of motivational items in the exploratory factor analysis in the main study

Motivational items	Cronbach α if item was deleted
Financial prospects	0.727
Perceived status of the field	0.724
Opportunities for higher studies or further specialization	0.728
Professionally challenging	0.745
Less stressful working conditions	0.716
Flexible working hours	0.715
Opportunity to settle down in urban areas	0.721
Influences from past experiences	0.728
Opportunity to do research	0.743
Interest in specialty	0.739
Family responsibilities	0.722
Preference to work in rural areas	0.755
Opportunity to be involved in patient care	0.743
Opportunity to teach	0.730
Less duration of work hours	0.720
Influenced by role models	0.734
Sufficient time for hobbies and personal interests	0.713
Family members in same specialty	0.726
Comfortable lifestyle	0.715
Sense of calling	0.739

Exploratory factor analysis was followed by extraction by principal component analysis with varimax rotation and Kaiser normalization.
