# Supplementary material for: Factors influencing the career preferences of medical students and interns: a cross-sectional, questionnaire-based survey from India
Source: J Educ Eval Health Prof. 2019 May 15;16:12. doi: 10.3352/jeehp.2019.16.12 (PMC6609296; doi:10.3352/jeehp.2019.16.12)
Supplement: Supplementary file 2 [file jeehp-16-12-app1.pdf]

Appendix 1. Questionnaire used in the study (PMCS Questionnaire version 4.0).

Perception of Medical students on Career Specialty (PMCS) Questionnaire

Instructions

The following questionnaire is designed to assess the attitude and perception of medical students on the choice of career specialty. Some general instructions for answering this questionnaire are:

1. There are a total of 12 items in this questionnaire. Please ensure that you answer ALL the items.
2. Answer the questions to the best of your understanding. AVOID discussion or clarifications about the questions and/or its options with your friends around.
3. Some questions have specific instructions on answering them; please READ them carefully. If you wish to change your answer for some reason, strike out the previous answer completely.
4. FOLLOW these instructions to create your participant unique ID which would have seven characters. Do not use any identifiers (name, number or batch) other than this ID anywhere in this questionnaire.
   The *first* character should indicate your *gender*. The *second and third* characters should indicate the date you were born. The *fourth and fifth* characters should indicate the year of birth. The *sixth* and *seventh* characters should indicate the *first alphabet of your parents' names* (father's name first).

For example, if you are a female born on 4th Feb 1995 and your parents' names are Rajkumar and Nisha, your participant unique ID will be F0495RN.

Fill participant unique ID in the box below:

Gender	Date of birth	Year of birth	First alphabet of father's name	First alphabet of mother's name

1. Indicate the year of joining Bachelor of Medicine and Bachelor of Surgery (MBBS): ________________________

2. Indicate the place you were brought up during most of your childhood:

State ________________ City________________ Town______________ Village________________

3. Indicate your board of education in higher secondary school (standard XII).

a) State________________ b) CBSE (Central Board of Secondary Education)

c) ISC (Indian School Certificate) d) Others

4. Do you have a family member who is a doctor or working in a profession related to the medical field?

a) Yes b) No

If Yes, indicate the relationship and nature of his/her work:

5. Are you planning to do a postgraduate degree after MBBS?

a) Yes b) No c) Not sure

i) If your response was Yes, where would you like to do your postgraduate degree after MBBS?

a) India b) Abroad c) Not sure

ii) If your response was No, state your reason:

6. Where would you prefer to work after completion of your postgraduate degree?

i. a) Urban areas b) Rural areas c) Not sure

ii. a) Mission hospital b) Tertiary care hospital (including Christian Medical College)
c) Own or private hospital d) Others, please specify:

7. Which of the following statements best describe your current state of mind, regarding

i. the awareness on the various career specialties available?
ii. the certainty of the course you want to pursue?
a) I am not aware of all career specialties available, and I am not certain of my choices
b) I am not aware of all career specialties available, but I am certain of my choices
c) I am aware of all career specialties available, but I am not certain of my choices
d) I am aware of all career specialties available, and I am certain of my choices

8. The broad medical specialty degrees (Doctor of Medicine [MD], Master of Surgery [MS], Diplomate in National Board [DNB]) that a doctor can choose after MBBS are listed below.

i) Indicate three specialties you would most likely choose (*numbers 1, 2, and 3 in the order of your preference*)
ii) Indicate three specialties you would least likely choose (*use cross [X]*)

Specialty	Choice	Specialty	Choice
Anatomy		Obstetrics & gynecology	
Anesthesia		Ophthalmology	
Biochemistry		Orthopedics	
Community medicine		Pathology	
Dermatology		Pediatrics	
Emergency medicine		Pharmacology	
ENT (ear, nose, or throat)		Physical medicine and rehabilitation	
Family medicine		Physiology	
Forensic medicine		Psychiatry	
General medicine		Radiodiagnosis	
General surgery		Radiotherapy	
Geriatric medicine		Respiratory medicine	
Microbiology		Transfusion medicine	
Nuclear medicine		Not sure	

9. On a scale of 1 to 5 (where, 1=not important and 5=very important), how would you rate the importance you would assign to each of the following factors for choosing a specialty?

Indicate your rating against each of the options even if you are not sure.

Factor	Rating	Factor	Rating
Financial prospects		Family responsibilities	
Perceived status of the field in society		Preference to work in rural areas	
Opportunities for higher studies or further specialization		Opportunity to be involved in patient care	
Professionally challenging field		Opportunity to teach	
Less stressful working conditions		Less duration of work hours	
Flexible working hours		Influence by role models	
Opportunity to settle down in urban areas		Sufficient time for hobbies and personal interests	
Influence from past experiences		Family members in the same specialty	
Opportunity to do research		Comfortable lifestyle	
Interest in the specialty		Sense of calling	
Others (Please specify):			

10. According to you, at what stage of MBBS will a medical student be able to clearly have an idea as to what specialty one is interested in?

a) At the time of joining MBBS b) End of I year c) End of II year
d) End of III year e) End of final year f) End of internship

11. Subsequent to completion of your specialty of choice, if you are given an opportunity to serve in rural or remote parts of the country, would you be willing to do so?

a) Yes b) No c) Not sure

12. On a scale of 1 to 5 (where, 1=not important and 5=very important), how would you rate the importance of having a specialty degree to serve in the rural/remote areas, in comparison to MBBS?

Mention your rating: __________
